# Crimean-Congo Hemorrhagic Fever, Spain, 2013–2021

**DOI:** 10.3201/eid2902.220677

**Published:** 2023-02

**Authors:** Helena Miriam Lorenzo Juanes, Cristina Carbonell, Begoña Febrer Sendra, Amparo López-Bernus, Alberto Bahamonde, Alberto Orfao, Carmen Vieira Lista, María Sánchez Ledesma, Ana Isabel Negredo, Beatriz Rodríguez-Alonso, Beatriz Rey Bua, María Paz Sánchez-Seco, Juan Luis Muñoz Bellido, Antonio Muro, Moncef Belhassen-García

**Affiliations:** Universidad de Salamanca, Salamanca, Spain (H. Lorenzo-Juanes, C. Carbonell, B. Febrer-Sendra, A. López-Bernus, A. Orfao, C. Viera Lista, M. Sánchez Ledesma, B. Rodriguez Alonso, B. Rey Bua, J.L. Muñoz Bellido, A. Muro, M. Belhassen-Garcia);; Instituto de Investigación Biomédica de Salamanca, Salamanca (H.M. Lorenzo Juanes, C. Carbonell, B. Febrer Sendra, A. López-Bernus, C. Vieira Lista, B. Rodriguez-Alonso, B. Rey Bua, M.P. Sánchez-Seco, A. Muro, M. Belhassen-Garcia);; Hospital Universitario de Salamanca, Salamanca (H.M. Lorenzo Juanes, C. Carbonell, M. Sánchez Ledesma, B. Rodríguez-Alonso, B. Rey Bua);; Hospital El Bierzo, Ponferrada, Spain (A. Bahamonde);; Centro de Investigación del Cáncer, Salamanca (A. Orfao);; Centro de Investigación Biomédica en Red de Cáncer, Madrid, Spain (A. Orfao);; Centro Nacional de Microbiologiia, Majadahonda, Spain (A.I. Negredo, M.P. Sánchez-Seco);; Red de Investigación Colaborativa en Enfermedades Tropicales, Madrid (A.I. Negredo, M.P. Sánchez-Seco, J.L. Muñoz Bellido);; Centro de Investigación Biomédica en Red en Enfermedades Infecciosas, Madrid (A.I. Negredo, M.P. Sánchez-Seco)

**Keywords:** Crimean-Congo hemorrhagic fever virus, hemorrhagic fever, viruses, tick-borne infections, zoonoses, Spain

## Abstract

Crimean-Congo hemorrhagic fever (CCHF) is a viral infectious disease for which distribution of the main vector, *Hyalomma* spp. ticks, is expanding. We analyzed all 10 cases of CCHF diagnosed in Spain during 2013–2021; case-patient median age was 56.5 years, and 7 were men. We identified CCHF virus genotypes III and V. Six case-patients acquired the infection in urban areas. Sixty percent of patients were infected in summer and 40% in spring. Two patients met criteria for hemophagocytic syndrome. Seven patients survived. The epidemiologic pattern of CCHF in Spain is based on occasional cases with an elevated mortality rate. Genotype III and, to a less extent also genotype V, CCHF circulates in humans in a common geographic area in Spain. Those data suggest that the expansion pathways are complex and may change over time. Physicians should remain alert to the possibility of new CCHF cases.

## Abstract 2

Physicians should be alert to the possibility of new cases, given the high pathogenicity of this virus.

Crimean-Congo hemorrhagic fever (CCHF) is a tickborne viral disease caused by the CCHF virus (CCHFV), a negative single-stranded RNA virus of the genus *Orthonairovirus* in the Nairoviridae family (*1*). CCHF is considered an emerging infectious disease because of the expanding distribution of its main vector, ticks of the genus *Hyalomma*. Consequently, CCHF is listed by the World Health Organization as one of the top-priority diseases for research and development in public health emergency contexts (https://www.who.int/activities/prioritizingdiseases-for-research-and-development-in-emergency-contexts) (*2*).

The spectrum of clinical manifestations of CCHF ranges from subclinical illness (including fever, headache, malaise, myalgia, sore throat, dizziness, abdominal pain, nausea, vomiting, conjunctivitis, and photophobia) (*3*) to acute infection with hemorrhage, multiorgan failure, and death (*4*). Laboratory findings are frequently remarkable, including leukopenia, thrombocytopenia, and elevated liver transaminases in serum (*5*). Some studies have suggested the relevance of the innate immune system in limiting the spread of the virus, but the specific mechanisms leading to asymptomatic versus severe disease remain unknown.

In recent years, the epidemiology of CCHFV has changed; climate change has been identified as one of the factors driving the circulation of the virus. CCHFV has been identified in Africa, Asia, and Europe, in territories located south of the 50th North parallel, the area inhabited by its main vector (*6*–*8*). CCHFV has caused major outbreaks in eastern Europe (*9*). In turn, CCHF is considered endemic in areas of southwestern Europe. 

Our group identified the first human cases in western Spain in summer 2013 (*10*–*13*). In Spain, the CCHFV genotype identified from patients in 2016 and 2018 belonged to the African genotype III, the European genotype V, and the Asian genotype IV where the group Africa 4 is placed (*10*,*12*,*13*). A strong clinical suspicion is required to obtain fast and accurate diagnosis, initiate supportive treatment if needed, and activate biosafety measures to prevent nosocomial transmission (*10*). Herein, we report on the clinical and epidemiologic pattern and the genotype of the virus identified in all patients with CCHF investigated in Spain from 2013 through May 2022.

The Clinical Research Ethics Committee of Investigation with Drugs of the Hospital Universitario de Salamanca (Salamanca, Spain) approved the study protocol (CEIMC PI 91 09/2017). All procedures described were carried out in accordance with the ethical standards described in the Revised Declaration of Helsinki of 2013. All clinical and epidemiologic data were anonymized.

## Methods

### Study Type and Sample Collection

We retrospectively analyzed records of all patients that had been diagnosed of CCHF in Spain during 2013–2022. Five cases were identified after searching PubMed for literature published during 2016–May 2022. Four cases were identified at Hospital Universitario de Salamanca (Salamanca, Spain) in 2020–2021. Another case was identified at Hospital del Bierzo (Ponferrada, Spain) in June 2021. All epidemiologic, clinical, and analytical parameters were recorded according to a predefined clinical protocol. For all cases, diagnosis of CCHF was confirmed at the National Microbiology Center of the Instituto de Salud Carlos III (Madrid, Spain).

### Phylogenetic Analysis

We aligned CCHFV sequences using ClustalW software (https://www.genome.jp/tools-bin/clustalw). We constructed the phylogenetic tree using a Tamura 3-parameter model based on sequences of the CCHFV small segment. We used the neighbor-joining method in MEGA X software version 10.2.5 (https://www.megasoftware.net) and based bootstrap confidence limits on 1,000 replicates.

### Statistical Analyses

We used the SPSS Statistics 25.0 (https://www.ibm.com/spss) for all statistical analyses. We calculated median (range) and mean values and their SDs for continuous variables; we used frequencies for categorical variables.

## Results

**Figure 1:**
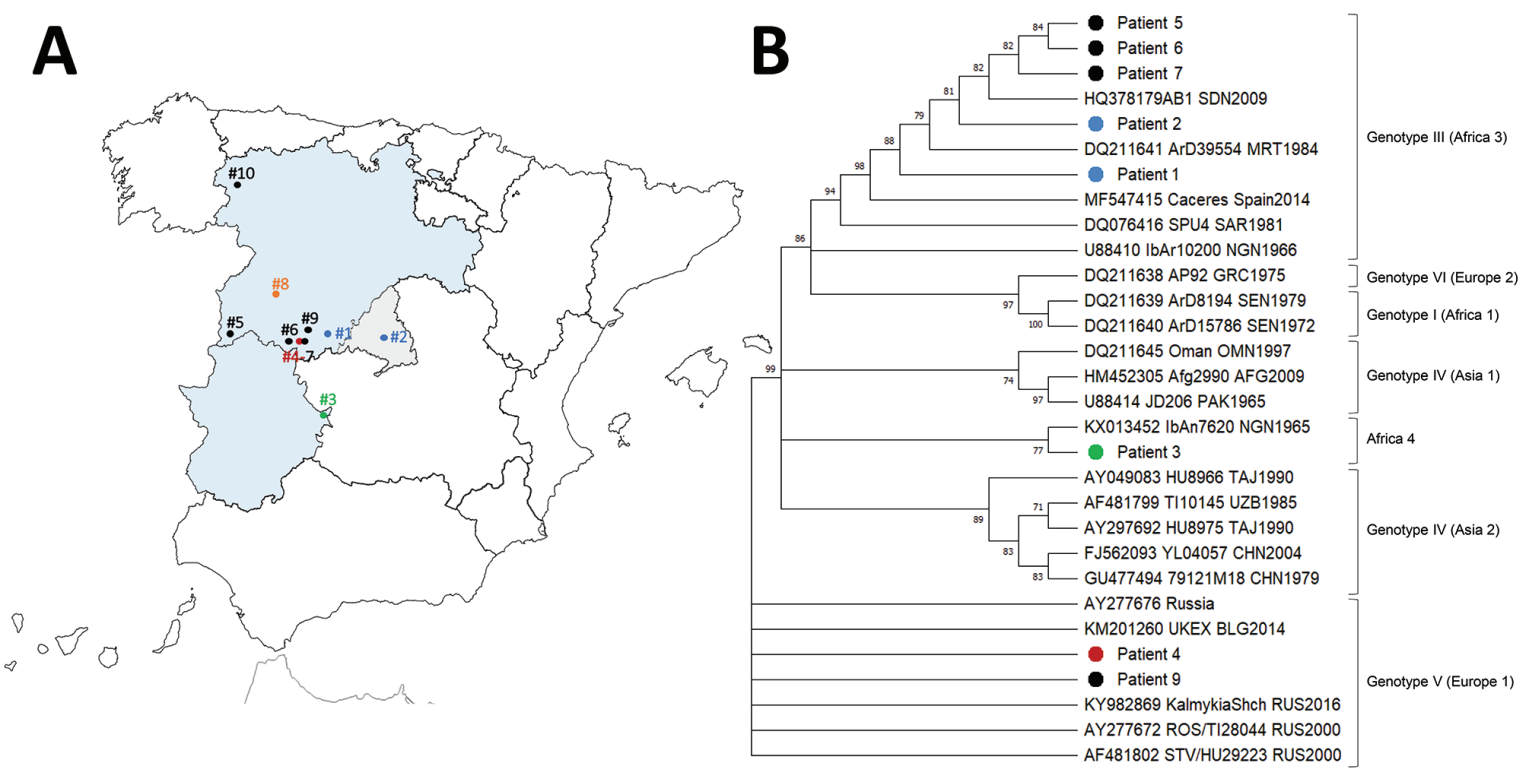
Locations of CCHF cases (A) and phylogenetic tree of CCHFV (B) in Spain, 2013–2021. Dots on the map indicate patients with a CCHF diagnosis in Spain: black dots indicate cases from this study, and colored dots indicate cases previously described. Two cases (patients 8 and 10) were not sequenced. The phylogenetic tree was constructed by the neighbor-joining method based on sequences of the small segment of the virus. The numbers on the right indicate bootstrap values for the groups; values <75 are not shown. Other sequences are listed by GenBank accession number, strain, geographic origin, and sampling year. Genotypes are indicated by Roman numerals according to Carroll et al. (*14*) with the equivalent clade nomenclature according to Chamberlain et al. (*15*) in brackets; I, West Africa (Africa 1); III, South and West Africa (Africa 3); IV, Middle East/Asia, divided into 2 groups (Asia 1/Asia 2); V, Europe/Turkey (Europe 1); VI, Greece (Europe 2). New lineage, Africa 4 described by Negredo et al. (12). CCHF, Crimean-Congo hemorrhagic fever; CCHFV, CCHF virus.

We extracted data for patients 1, 2, 3, 4, and 8 from published papers (*10*–*13*). In turn, we conducted retrospective analysis on the medical records of all other patients with CCHF identified from Hospital Universitario de Salamanca and Hospital del Bierzo; we recorded demographic patient data, case history, symptoms, clinical signs, laboratory results, and outcomes for each patient (Table 1). Patient median age was 56.5 years (range 30–74 years); 7 were men and 3 women. Six patients had been infected in urban areas. The distribution of cases during the year was as follows: 1 case in April, 1 in May, 2 in June, 2 in July, and 4 in August (Figure 1). 

**Table 1:** Main epidemiologic data of patients with Crimean-Congo hemorrhagic fever, Spain, 2013–2021*

Characteristic	Patient no. and source									
	1 (*10*)	2 (*10*)	3 (*12*)	4 (*13*)	5	6	7	8 (*11*)	9	10
Age, y	62	50	74	53	70	54	69	32	59	30
Sex	M	F	M	M	M	M	M	F	M	F
Rural location	No	No	No	Yes	Yes	Yes	Yes	No	Yes	No
Date	2016 Aug	2016 Aug	2018 Jul	2018 Aug	2020 Jun	2020 Jul	2020 Aug	2013 May	2021 Apr	2021 Jun
Risk factors†	Leisure	Nurse	Hunting	Ag	Ag	Ag	Leisure	Leisure	Ag	Leisure
Comorbidities	HTN, OSA	None	None	Hepatic steatosis, active drinker	Tongue cancer	TB, brucellosis, active drinker	HTN	None	Diabetes mellitus, dyslipemia	Diabetes mellitus
Bakir scale at admission	7	0	7	6	6	4	8	5	2	5
Outcome	Died	Good	Died	Good	Good	Good	Died	Good	Good	Good

*Source is indicated if other than this study. Ag, agriculture; HTN, hypertension; OSA, obstructive sleep apnea. †Risk factors include high-risk occupations; agriculture includes shepherding activities.

**Figure 2:**
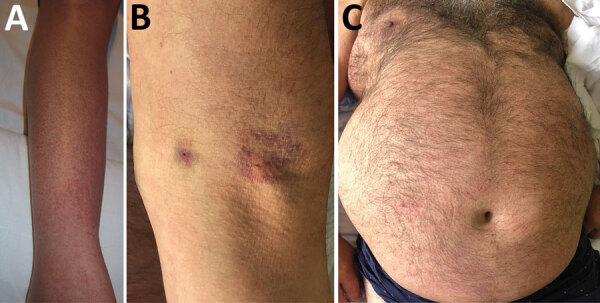
Images of patients in study of Crimean-Congo hemorrhagic fever, Spain, 2013–2021. A) Details of a slightly purpuric rash on the leg of patient 8. B) Ecchymosis on the arm of patient 5. C) Mild rash on the chest of patient 9.

**Figure 3:**
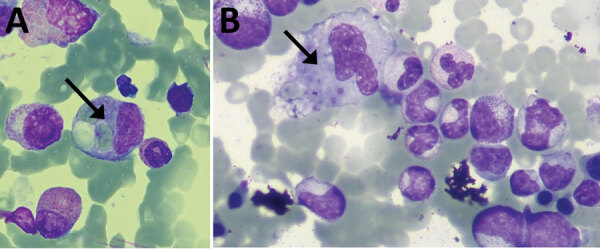
Bone marrow biopsy findings from patient 5 (A) and patient 9 (B) in study of Crimean-Congo hemorrhagic fever, Spain, 2013–2021. Arrows indicate macrophages with hemophagocytosis phenomena of red blood cells and platelets.

Eight of the 10 patients reported tick bites (Table 2). The mean +SD time from the bite to the onset of symptoms was 5.1 + 3.4 days (range 2–12 days). The median duration between the onset of symptoms and hospital admission was 5.1 + 3.1 days (range 2–12 days). All case-patients had sought care for fever and exanthema with a mean duration of 5.2 + 1.64 days (Figure 2). Eight patients had muscle soreness; 4 patients had diarrhea, and 4 had vomiting, nausea, or both. Three case-patients (5, 6, and 9) underwent bone marrow biopsy; 2 of them, patients 5 and 9, had hemophagocytosis, which fulfilled the criteria for hemophagocytic syndrome (Figure 3). Ferritin serum level was elevated in 7 patients.

**Table 2:** Main clinical and laboratory data of patients with Crimean-Congo hemorrhagic fever, Spain, 2013–2021*

Characteristic	Patient no. and source									
	1 (*10*)	2 (*10*)	3 (*12*)	4 (*13*)	5	6	7	8 (*11*)	9	10
Main clinical data										
Tick bite	Y	N	Y	N	Y	Y	Y	Y	Y	Y
First symptom	Fever	Fever	Fever	Fever	Fever	Fever	Fever	Fever	Fever	Fever
Fever duration, d	4	5	6	6	9	5	4	4	4	5
Days from first symptom to admission	3	2	4	5	9	7	3	2	4	12
Digestive symptoms	Y	Y	Y	N	Y	N	Y	Y	Y	Y
Any bleeding	Y	Y	Y	N	Y	N	Y	Y	N	Y
Laboratory data†										
Hemoglobin, g/dL	13.4	13.9	13.5	14.1	14.6	15.5	13.4	14.4	17	17
Leukocytes, × 10^3^ cells/mm^3^	13.9	6.2	10.7	3.1	2.4	2.3	5.5	1.5	2.8	11.1
Neutrophils, %	85.5	83	90	62	33	66.4	69	63	68.5	90
Lymphocytes, %	7.9	10.2	5	27	38	26.1	25	31	24.4	4
Platelets, × 10^3^/mm^3^	30	174	229	41	44	32	7	44	76	159
Glucose, mg/d	80	102	83	135	110	134	280	106	116	491
Creatinine, mg/dL	1.69	1.24	0.83	1.33	0.92	0.75	4.8	0.67	0.85	1.1
CRP, mg/L	87.6	2.9	ND	15.2	0.3	0.65	3.72	0.6	0.55	52
AST, U/L	203	24	20	347	273	273	1,305	494	107	72
ALT, U/L	88	37	9	161	281	135	347	171	141	70
Ferritin, ng/mL	ND	ND	>40,000‡	15,718	34,044	28,393	60,000	ND	7,878	1,147
Bilirubin, mg/dL	0.9	0.5	0.5	0.7	0.43	0.35	1.4	0.29	0.58	ND
GGT, U/L	ND	ND	ND	425	272	132	1,420	77	136	ND
ALP, U/L	ND	ND	ND	103	84	59	239	58	72	91
LDH, U/L	ND	ND	172	721	358	589	2,311	1,085	341	272
Triglycerides, mg/dL	ND	ND	ND	ND	ND	407‡	ND	ND	164‡	ND
Prothrombin time, s	18.1	15.6	10.7	10.2	10	12	13	12	11	ND
Prothrombin activity, %	52.8	62	104	106	123	99	86	81	102	84
Partial thromboplastin time, s	18.1	48.7	26.2	43.8	30.2	52.7	61.4	128	ND	29
Functional fibrinogen, mg/dL	ND	265.9	320	605	281	304	156	141	272	325
D-dimer, ng/mL	ND	35,200	ND	ND	ND	1.3	5.5	3.48	ND	ND
Genotype	III	III	IV	V	III	III	III	ND	V	ND
Treatment	DOX; support	DOX; Ribavirin ev/orally for 9 d	DOX; support	DOX	DOX; ribavirin orally for 10 d§	DOX; ribavirin orally for 10 d§	DOX support	DOX; support	DOX	DOX; support
Length of stay, d	9	23	8	6	22	9	2	17	9	8

*Source is indicated if other than this study. ALP, alkaline phosphatase; ALT, alanine aminotransferase; AST, aspartate aminotransferase; CRP, C-reactive protein; DOX, doxycycline; GGT, gamma-glutamyl transferase; LDH, lactate dehydrogenase; ND, no data. †Analysis upon admission or during the first 24 hours. ‡Analysis performed during hospital admission. §In accordance with World Health Organization guidelines.

All patients received antimicrobial treatment with doxycycline while hospitalized. In addition, 5 patients received supportive treatment, 4 patients had taken treatment for bleeding, and 3 patients received ribavirin. None of those receiving antiviral treatment died; however, the sample size was small.

The mean +SD score on the Bakir prognostic scale (*16*) was 5.0 + 2.3. Seven patients survived with full recovery, whereas the other 3 died. Those 3 patients who died had the highest scores on the Bakir scale (1 patient scored 8 and the other 2 scored 7).

In most cases (patients 1, 2, 5, 6, and 7), disease was caused by CCHFV genotype III (Africa 3). Patient 3 had a new lineage, Africa 4 (Figure 1) within genotype IV. Isolates from patients 4 and 9 belonged to genotype V (Europe 1). We did not identify the genotype for patients 8 and 10. Of note, genotypes III and V were found to circulate in the same geographic area. We deposited the sequences into GenBank under accession nos. KY492290 (patient 1), KY492289 (patient 2), MN689739 (patient 3), ON227355 (patient 4), OP776634 (patient 5), OP776632 (patient 6), OP776631 (patient 7), and OP776633 (patient 9). 

## Discussion

We describe the demographic, epidemiologic, clinical, and laboratory features of all 10 cases of CCHFV reported in Spain since 2013, when the causative agent was first discovered in this country (*11*). Our findings have updated the knowledge of CCHFV in Spain to show the pattern during the period 2013–2021 in southern Europe. Half (5/10) of the cases we described in this article were treated at Hospital Universitario de Salamanca *(**10*–*13*). CCHFV was found in western Spain in 2010 in ticks (*Hyalomma lusitanicum*) feeding on wild animals in the province of Caceres (*17*).

Studies carried out in the same period and the same geographic areas in Spain in healthy donors (*18*) showed a serologic prevalence of past infection of ≈1.16% and in patients who sought emergency care for febrile syndrome (*13*) of ≈2.22%. Altogether, these results suggest that CCHF is underdiagnosed in this region. However, the high frequency of asymptomatic patients, which exceeds 88% in some studies, might also account for such differences (*3*,*4*).

Spain’s geographic proximity to Africa is a risk factor for continuous entry of CCHFV. Its favorable climate, the wide presence of the vector involved in transmission, the variety of vertebrate animals that can act as amplifying hosts, and its location along the path of transit for migratory birds from CCHFV-endemic areas all increase the risk for spread of the virus.

Most infections in this study occurred in spring and summer in rural areas of central-western Spain. Of the cases that occurred in the southern part of the autonomous community of Castile and León, likely causes are specific climatic features (e.g., temperature, humidity), geographic conditions, flora and wildlife, the animal husbandry sector, and increased contact with animals and ticks. In this regard, field studies have confirmed that these areas are at risk for CCHF because of the abundance of *H. lusitanicum* ticks, the presence of CCHFV in the specimens collected, together with the high prevalence observed in wild and domestic animals in these and other areas of the country (*17*,*19*–*22*).

The epidemiologic pattern we report for Spain resembles that of countries such as Greece and Kosovo (*23*,*25*), with few and occasional cases, and clearly differs from the epidemiologic evolution of countries such as Turkey (*25*), which has a marked and progressive increase in cases since its earliest recorded case in 2002. Those distinct epidemiologic evolution profiles might be related to differences in farming and sheepherding activities, as well as the specific climate conditions; in Turkey, a notable and specific risk factor is living at altitudes >836.5 m (*26*). We noted the possibility of secondary transmission of CCHF to healthcare workers, particularly during accidental contact such as resuscitation of severely ill patients, and the need for strict rules and protocol for handling potential secondary cases (*10*). 

Clinical findings in the patients we reviewed revealed that the most common symptoms were fever, exanthema, and myalgia. However, we also noted findings of bleeding (7/10 cases) at higher frequencies than those reported previously (*25*). Two patients with CCHFV experienced hemophagocytic syndrome with hemophagocytosis in the bone marrow. Hemophagocytic syndrome is a rare and severe disease characterized by fever; hepatosplenomegaly; cytopenia; elevated ferritin, lactate dehydrogenase and triglyceride levels; and hemophagocytosis in the bone marrow. Clinical and biologic symptoms of hemophagocytic syndrome are caused by cytokines secreted by T-lymphocytes and macrophages. A main challenge in patients with hemophagocytic syndrome is its diagnosis, which must meet well-established criteria (*27*). The relationship between CCHFV and hemophagocytic syndrome has been previously described (*28*), but unlike in those studies, the patients in our review did not experience serious bleeding episodes. However, high levels of serum ferritin in patients who underwent analytical determination suggest a higher prevalence of hemophagocytic syndrome than previously described; further research is needed to elucidate the specific mechanisms involved.

All patients received doxycycline accompanied by other antimicrobial drugs, possibly because of initial suspicion of rickettsiosis. Five patients required intensive care treatment; 3 of them died. Ribavirin was prescribed to 3 patients, who all recovered and survived. Despite the potential benefit of ribavirin, the small number of patients makes it difficult to draw conclusions regarding its effectiveness for treating CCHF patients. Furthermore, a recent Cochrane meta-analysis was unable to confirm the potential benefit of ribavirin in CCHFV-infected patients (*29*).

In Spain, where most CCHF patients have been diagnosed since 2018, the fatality rate of CCHF was as high as 30%. Of note, those 3/10 patients who died showed the highest Bakir-scale scores (>7) at admission. Previous studies have shown that in this viral infection transmitted by ticks, regional differences in mortality rates may be related to factors including the availability of advanced medical care facilities, faster diagnosis because of a better surveillance system that enables early detection of cases with mild to moderate clinical findings, the routes of acquisition of the infection, and the genotype of the virus. In Turkey, which has a CCHF mortality rate of ≈5%, the most common strain is homologous to the strain detected in Russia and Kosovo, whereas in Spain, the most common strain is the Africa III. Two of our cases infected with strain V have had good outcomes described; cases of genotypes III and V have been detected in the same area.

Previous studies indicated that birds are involved in the transmission of the Africa genotype III virus (*30*). Domestic animals such as pigs have been imported from countries in eastern Europe, indicating a possible relationship with the CCHF epidemiology of European strains, particularly those of genotype V. Of interest, a strain of CCHFV was detected in ticks in Spain (*21*). From a clinical point of view, genotypes III and IV have been associated with more deaths than genotype V in our cases, although the number of patients still remains very limited.

In Spain, circulation in wild animals of 3 different genotypes (III, IV, and V) of CCHFV has been demonstrated, even in the same geographic area; genotype III was the most prevalent. Those data suggest that the expansion pathways of the different CCHFV genotypes in Spain are complex and coincide over time; further studies are needed to clarify the dissemination of CCHFV in southern Europe. In addition, our results revealed a complex epidemiologic pattern in Spain in which uncommon CCHF cases were associated with high mortality rates. Thus, although the risk is considered low, hospital doctors and general practitioners should be alert to the possibility of new CCHF cases, given the high pathogenicity of CCHFV. A detailed medical history of the patient, including travel history and possible risk factors, is critical for fast diagnosis and appropriate adoption of therapeutic measures for timely control of the infection.
